# A Meta-Epidemiological Analysis of Sex Disparities in Hepatology Clinical Trials

**DOI:** 10.1016/j.cgh.2025.07.017

**Published:** 2025-07-22

**Authors:** ANNA M. GOEBEL, EMILY SHENG, PEDRO OCHOA-ALLEMANT, NOSHEEN REZA, NADIM MAHMUD

**Affiliations:** Department of Medicine, Perelman School of Medicine, University of Pennsylvania, Philadelphia, Pennsylvania; Department of Medicine, Perelman School of Medicine, University of Pennsylvania, Philadelphia, Pennsylvania; Division of Gastroenterology and Hepatology, Perelman School of Medicine, University of Pennsylvania, Philadelphia, Pennsylvania; Division of Cardiovascular Medicine, Perelman School of Medicine, University of Pennsylvania, Philadelphia, Pennsylvania; Division of Gastroenterology and Hepatology, Perelman School of Medicine, University of Pennsylvania, Philadelphia, Pennsylvania, *and* Leonard Davis Institute of Health Economics, University of Pennsylvania, Philadelphia, Pennsylvania, *and* Center for Clinical Epidemiology and Biostatistics, Department of Biostatistics, Epidemiology & Informatics, Perelman School of Medicine, University of Pennsylvania Philadelphia, Pennsylvania, *and* Department of Medicine, Corporal Michael J. Crescenz VA Medical Center, Philadelphia, Pennsylvania

Clinical trials have historically favored male participation leading to guidelines that may overlook sex-based differences.^1^ Although disparities vary by specialty (women are underrepresented in cardiology, nephrology, and hematology, but more equally represented in dermatology and infectious diseases), liver trials remain largely unexamined.^2^ This study aimed to evaluate global trends in female inclusion in randomized controlled trials (RCTs) for liver diseases.

In this meta-epidemiologic observational study we used a Medical Subject Headings (MeSH) search string in the PubMed database (Supplementary Methods) to identify English language RCTs on adult liver disease published between January 2013 and December 2024. PRISMA guidance was applied where relevant (Supplementary Table 1). Studies were included if they focused on chronic liver disease other than metabolic dysfunction-associated steatotic liver disease (non-MASLD CLD), MASLD CLD, liver cancer, and a composite of cirrhosis and/or liver transplantation. Exclusion criteria included missing sex data and trials exclusively enrolling 1 sex because of study-specific factors. Two authors (AMG, ES) manually screened and extracted data on study region, sample size, and female enrollment.

Enrollment benchmarks were derived from annual National Health and Nutrition Examination Survey data to estimate the proportion of women with non-MASLD CLD and MASLD and from United Network for Organ Sharing data to determine the proportion of women waitlisted for liver transplantation as a proxy for the cirrhosis/liver transplant domain, and the proportion of waitlisted women with liver cancer.

We used Kruskal-Wallis and chi-square tests and evaluated trends using weighted scatterplots and linear regressions. Participation-to-prevalence ratios (PPRs) were calculated and plotted over time to assess changes in representation relative to benchmarks. PPRs between 0.8 and 1.2 indicate adequate representation.^3^ To examine regional and domain-specific variation in PPRs, we fit a weighted linear regression with a region-by-domain interaction, adjusting for secular trends.

A total of 1593 RCTs were included. The median sample size varied by domain (eg, median 118 for non-MASLD CLD vs 69 for MASLD; *P* < .001; Supplementary Table 2). Proportions of women enrolled differed significantly by domain (eg, 47% MASLD vs 18% liver cancer; *P* < .001). Most studies were performed solely at sites in Asia (36.3%), followed by Europe (19.5%) and the United States (16.0%).

Weighted study proportions of women in RCTs across study domains are shown in Figure 1. For non-MASLD CLD, and cirrhosis/liver transplant study domains, the fitted proportion of women enrolled in trials was visually lower than expected benchmarks during each study year in descriptive analysis (Figure 1A and C), whereas proportions seemed more comparable in MASLD and liver cancer trials (Figure 1B and D). Weighted linear regression revealed a significant decline in female enrollment in non-MASLD CLD trials (beta, −0.004; 95% confidence interval, −0.007 to −0.001; *P* = .006; Figure 1A) and a significant increase in MASLD trials (beta, 0.006; 95% confidence interval, 0.001–0.012; *P* = .025; Figure 1B), but no statistically significant trends in cirrhosis/liver transplant or liver cancer trials (each *P* > .05; Figure 1C and D). Over time, women were consistently underrepresented in non-MASLD CLD trials, with PPRs as low as 0.61 in 2016, and intermittently underrepresented in cirrhosis/transplant and liver cancer trials (Figure 1E). In contrast, PPRs in MASLD trials remained balanced (0.8–1.2) throughout the decade of observation.

In a weighted linear regression model accounting for secular trends, there was a statistically significant interaction between trial region and study domain in a model with PPR as the outcome (*P* = .012; Figure 1F). For instance, women were underrepresented in non-MASLD CLD studies in the United States but not in other regions. Women were adequately represented in MASLD trials across all regions.

In this study of global RCTs, women were consistently underrepresented in non-MASLD CLD but adequately represented in MASLD trials; there was intermittent underrepresentation or borderline representation of women in cirrhosis/liver transplant and liver cancer trials. When analyzing sex-based differences in trial enrollment, it is critical to consider the disease burden within the general population, which we accounted for in this study using national epidemiology benchmarks from National Health and Nutrition Examination Survey and United Network for Organ Sharing registries.

Women may be underenrolled in non-MASLD CLD trials because of sex-specific eligibility criteria, biologic differences, systemic bias, and barriers including low referral rates and financial or cultural obstacles.^4^ Sex-specific exclusion criteria include those related to female hormones, pregnancy, and comorbidities.^5,6^ A National Institutes of Health report also cited logistical challenges, including transportation and caregiving duties.^7^

Systemic biases may stem from enrollment strategies and setting. For example, women were well-represented in MASLD studies, possibly because of high disease prevalence and recruitment from primary care settings, and reasonably well-represented in cirrhosis/transplant and liver cancer trials, where enrollment is often driven by disease severity and standardized listing criteria. In contrast, women were underrepresented in non-MASLD CLD trials, where disparities in early diagnosis, referral patterns, and specialty clinic recruitment may have limited their inclusion.

Addressing the underrepresentation of women in RCTs requires patient-level strategies including flexible scheduling, expense reimbursement, caregiver support, and culturally sensitive communication. Investigator-level solutions include avoiding overly restrictive selection criteria; incorporating sex-specific analyses; and increasing engagement with female researchers, especially in leadership and first author roles, which is correlated with higher female enrollment.^8,9^ At the policy level, enforcing sex-stratified enrollment targets, as with the National Institutes of Health Revitalization Act of 1993, can promote equitable inclusion.^10^

This study has limitations. Reported sex in trials reflects biologic sex at birth, not gender identities. Our analysis included only English-language RCTs and disease prevalence was based on National Health and Nutrition Examination Survey/United Network for Organ Sharing data, limiting geographic generalizability. Finally, PPR may not capture nuances, such as disease severity, health care access, or structural barriers to enrollment. The decline in trials during 2021–2022 likely reflects COVID-19 disruptions, whereas the decline in non-MASLD trials reflects reduced demand for hepatitis C virus studies. The search was limited to PubMed without librarian support, which may have missed relevant studies in other databases. Grouping cirrhosis and transplant trials may have masked differences between these populations.

In conclusion, women were consistently underrepresented in non-MASLD CLD RCTs and intermittently underrepresented in cirrhosis/transplant and liver cancer studies, with adequate representation in MASLD RCTs. Enrollment disparities may limit the generalizability of trial findings and access to new therapies for women. Future work should explore strategies to improve enrollment equity.

## Supplementary Material

Supplementary Methods

Supplementary Material

Note: To access the supplementary material accompanying this article, visit the online version of *Clinical Gastroenterology and Hepatology* at www.cghjournal.org, and at https://doi.org/10.1016/j.cgh.2025.07.017.

## Figures and Tables

**Figure 1.**
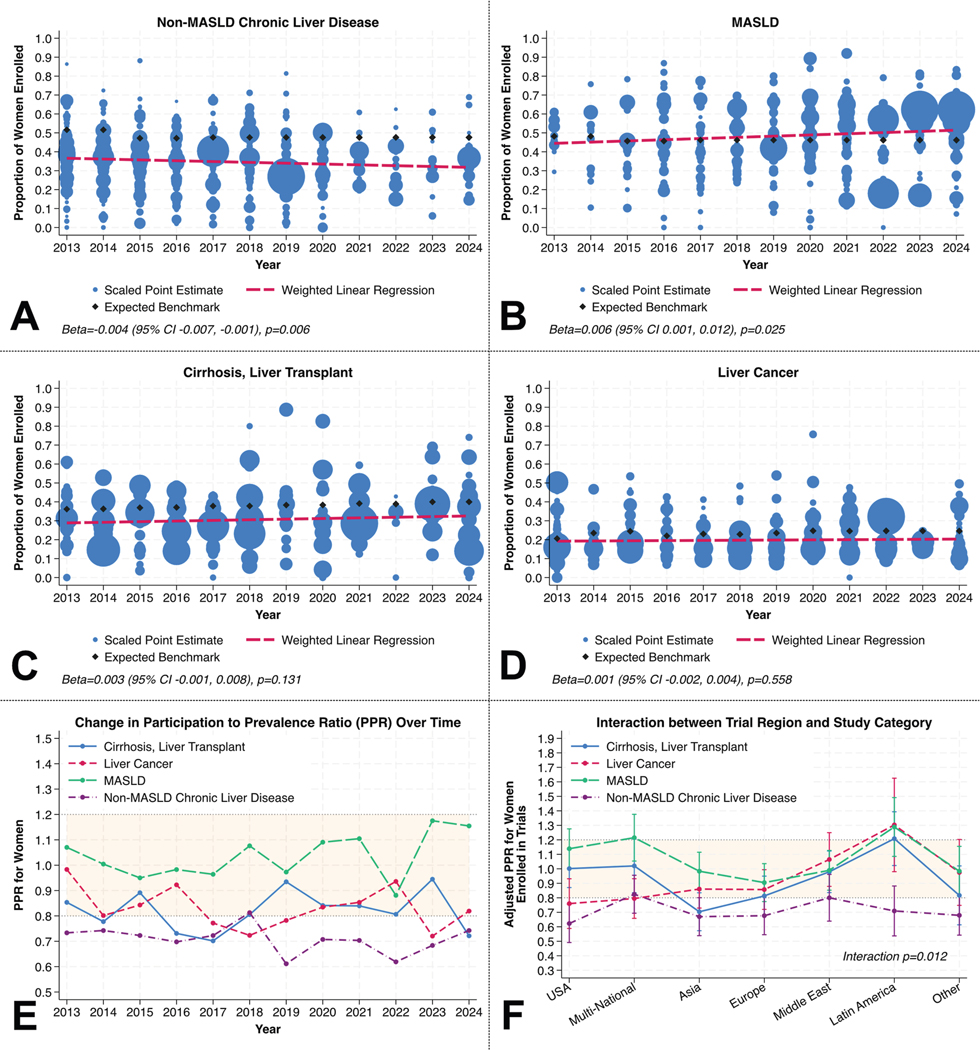
Weighted trends in proportion of women enrolled in trials for (*A*) non-MASLD chronic liver disease, (*B*) MASLD, (*C*) cirrhosis/transplant trials, (*D*) liver cancer trials, (*E*) associated changes in PPRs, and (*F*) difference in adjusted PPRs by trial region and study category. CI, confidence interval.

## Abbreviations used in this paper:

CLD: chronic liver disease
MASLD: metabolic dysfunction-associated steatotic liver disease
PRR: participation to prevalence ratio
RCT: randomized controlled trial
